# Inner Retinal Dysfunction in the Autosomal Recessive Spastic Ataxia of Charlevoix-Saguenay

**DOI:** 10.3389/fneur.2017.00523

**Published:** 2017-10-12

**Authors:** François-Xavier Borruat, Graham E. Holder, Fion Bremner

**Affiliations:** ^1^Hôpital Ophtalmique Jules-Gonin, Lausanne, Switzerland; ^2^Moorfields Eye Hospital, London, United Kingdom; ^3^University College London Institute of Ophthalmology, London, United Kingdom; ^4^The National Hospital for Neurology and Neurosurgery, London, United Kingdom

**Keywords:** autosomal recessive spastic ataxia of Charlevoix-Saguenay, Charlevoix-Saguenay, erg, On− bipolar cells, electronegative electroretinography, foveal hypoplasia, peripapillary retinal nerve fiber layer thickening, optical coherence tomography

## Abstract

The autosomal recessive spastic ataxia of Charlevoix-Saguenay (ARSACS) is associated with structural retinal abnormalities either directly visible on funduscopy or revealed by optical coherence tomography (OCT). Most patients with ARSACS have a whitish peripapillary appearance corresponding to a thickening of the peripapillary retinal nerve fiber layer. OCT has also shown an absence of the physiological foveal depression. Abnormal electroretinography (ERG) has previously been reported in only two cases, without further details. This report describes a patient with ARSACS in whom careful full-field ERG revealed dysfunction of the retinal On− bipolar cells with sparing of photoreceptor function. This is the first report of inner retinal dysfunction in ARSACS.

## Introduction

The autosomal recessive spastic ataxia of Charlevoix-Saguenay (ARSACS), related to mutation in the *SACS* gene on chromosome 13 (1), is mainly characterized by progressive ataxia of childhood onset and limb spasticity. The first description of ARSACS also reported the presence of “retinal striations” (2). Thickening of the peripapillary retinal nerve fiber layer (RNFL) is visible on fundus examination and appears pathognomonic of ARSACS; it has not been reported in other forms of hereditary ataxia (3). However, there is phenotypic variability as not all patients with ARSACS exhibit peripapillary RNFL thickening (4, 5). Although two cases have been described as having asymptomatic retinal dysfunction on electroretinography [ERG (2, 6)], visual performance has otherwise been reported to be normal (2, 7).

This report presents the results of detailed retinal imaging, ERG, and brain imaging in a patient with genetically confirmed ARSACS.

## Case Report

A 21-year-old man was referred for investigation of progressive visual loss in the setting of slow progressive ataxia since age 2. Neurological examination revealed a slight dysarthric speech, a moderate spastic paraparesis with brisk osteotendinous reflexes, a moderate dysmetria in four limbs, and a spastic and ataxic gait without sensory disturbance. Genetic testing for Friedreich ataxia and spinocerebellar ataxia type 1, 2, 3, 6, 7, and 17 were negative. Serum vitamin E level was normal. A diagnosis of spinocerebellar ataxia of unknown etiology was given.

Distance visual acuity was 4/10 in either eye whereas near vision was better at 6/10 RE and 8/10 LE. Color vision was normal on Ishihara pseudoisochromatic plates, and computerized static visual fields were full bilaterally. Central keratoconus was responsible for the decreased visual acuity.

**Figure 1:**
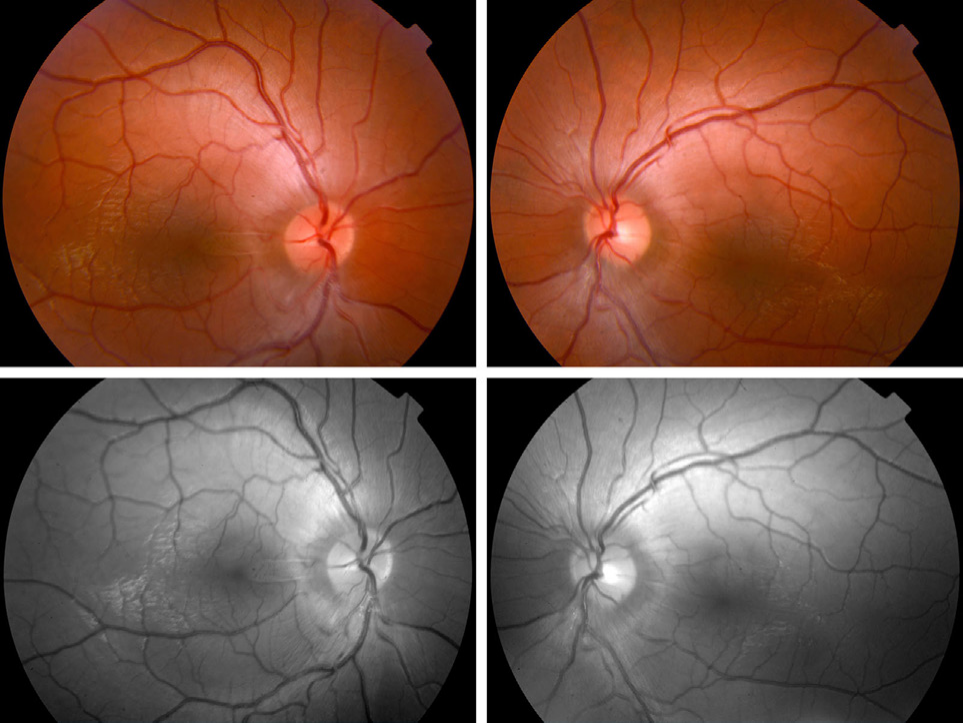
Fundus photographs. Top—fundus examination reveals a whitish striated appearance of the peripapillary retina. Note that the disk margin is clear, without dilation of the optic disk capillaries or hemorrhages, and that the retinal abnormality begins distant from the optic disk margin. Bottom—red free photographs show better the appearance of the peripapillary retinal nerve fiber layer.

**Figure 2:**
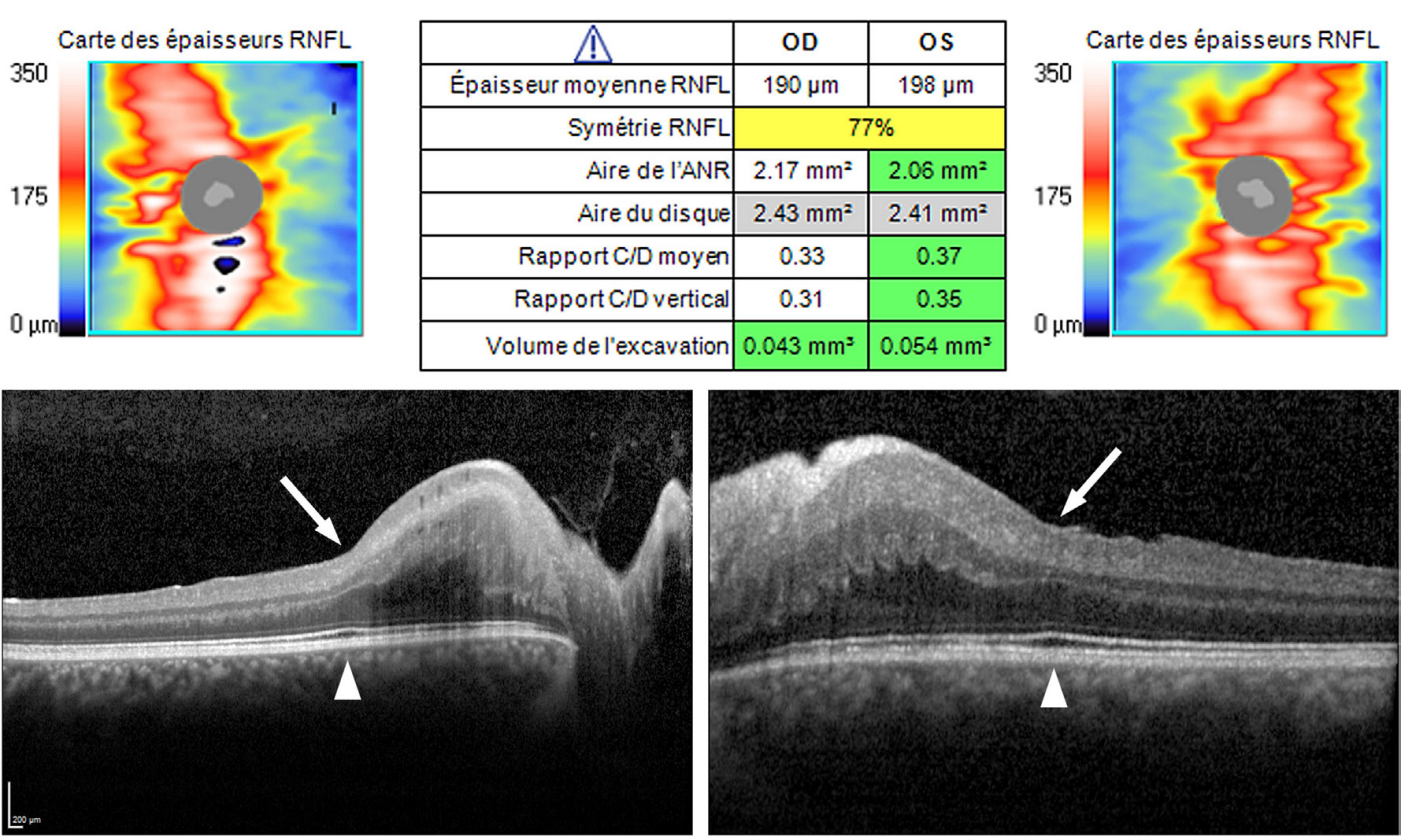
Optical coherence tomography (OCT). Top—OCT (Cirrus, Carl Zeiss Meditec, Inc., Germany). Peripapillary retinal nerve fiber layer (RNFL) was regularly thickened in both eyes (190 µm RE, 198 µm LE; norm 85–110 µm), in all clock hours, with the preservation of the “double hump” appearance (i.e., thicker RNFL superiorly and inferiorly). Bottom—OCT (Spectralis, Heidelberg Engineering GmbH, Germany). A high definition horizontal line through the fovea shows the absence of the normal physiological foveal depression in both eyes (arrow), despite the outer retinal ellipsoid layer exhibiting normal thickening at the fovea (arrowhead).

**Figure 3:**
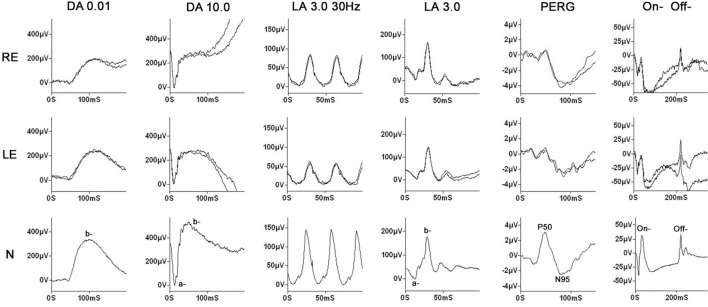
Electrophysiology. Full-field electroretinography (ERG) was performed using gold foil electrodes. The labeling is conventional and reflects the adaptive state of the eye [dark-adapted (DA) and light-adapted (LA)] and the flash strength (in cd·s/m^2^). The data from a representative normal subject are shown in the lower row for comparison. Dim flash DA ERGs (DA 0.01) show no definite abnormality; the bright flash DA ERGs (DA 10.0) have a negative (electronegative) waveform with a normal a-wave, confirming normal photoreceptor function, but a lower amplitude b-wave in keeping with inner retinal dysfunction. The 30 Hz flicker ERG is bilaterally delayed, and although the single flash LA ERG (LA 3.0) shows no definite abnormality, photopic long duration stimulation, to separate the function of the cone On− and Off− pathways, shows a markedly negative On− response (reduced b:a ratio). The findings overall indicate generalized inner retinal dysfunction in both rod and cone systems, probably predominantly involving the On− pathways. The pattern electroretinogram (PERG) is bilaterally subnormal in keeping with the optical degradation introduced by keratoconus.

Fundus examination revealed a striated whitish peripapillary appearance with a normal optic disk in both eyes (Figure 1). A significant thickening of the peripapillary RNFL was demonstrated by optical coherence tomography (OCT). Macular OCT showed absence of the normal foveal depression in both eyes (Figure 2). Fluorescein and indocyanine green retinal angiography were normal. Full-field ERG was performed to incorporate ISCEV standards (8). Bright flash dark-adapted ERG had an electronegative waveform (i.e., the b-wave amplitude was lower than the a-wave amplitude), in keeping with inner retinal rod system dysfunction (Figure 3). Photopic 30 Hz flicker ERG was mildly delayed bilaterally, and there was reduction in the b:a ratio of the On− response when On−/Off− response recording was performed.

**Figure 4:**
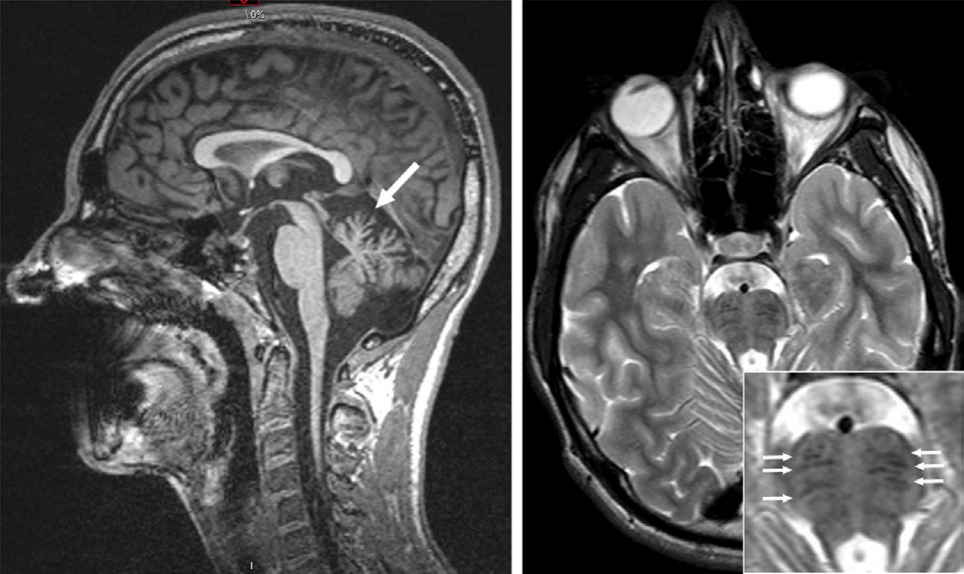
Magnetic resonance imaging (3 T). Left—sagittal cut, T1-weighted sequence revealing cerebellar vermis atrophy (arrow). Right—axial cut, T2-weighted sequence revealing bilateral and symmetrical hypointense linear lesions in the pons (small arrows in insert).

Magnetic resonance imaging revealed atrophy of the cerebellar vermis (Figure 4) and symmetrical linear hypointense striations on either side of the pontine midline on T1-weighted sequences (Figure 4). The combination of an atrophy of the superior cerebellar vermis with the presence of linear hypointense striated lesions in the pons on T2 or Flair sequences has been reported by several authors in ARSACS patients (9, 10). Our patient also exhibited mild generalized cerebral atrophy with slight dilation of the posterior ventricules and thinning of the corpus callosum. Genetic studies showed the presence of a homozygous point deletion (c6078) in the *SACS* gene confirming the diagnosis of ARSACS.

## Comment

This report provides a comprehensive description of retinal structure and function in a patient with mutationally confirmed ARSACS syndrome. Results of MRI were also suggestive of ARSACS.

The patient was initially referred for investigation of visual acuity reduction, which was actually due to undiagnosed bilateral keratoconus. Interestingly, the genes for ARSACS and keratoconus are both on chromosome 13, but genetic studies failed to disclose an abnormality at the location of the keratoconus gene.

The first report of ARSACS described “striking and markedly increased visibility of the retinal nerve fibers, mainly in the papillomacular bundle area” (2). The exact nature of the peripapillary RNFL thickening has been debated, but OCT findings suggest hypertrophy of the RNFL rather than myelination of RNFL (11, 12). The specificity of the peripapillary RNFL thickening is 100%, but sensitivity is lower with some patients, particularly those of Japanese origin, being reported not to exhibit such peripapillary RNFL thickening, either by fundus or by OCT examination (4, 5). OCT examination has also recently revealed an absence of the physiological foveal depression (7, 11–13). However, despite these structural abnormalities, visual dysfunction in ARSACS (using standard tests of visual acuity, visual field, and color vision) has not been previously reported. The present patient had visual acuity loss due to previously undiagnosed keratoconus, seemingly unrelated to ARSACS.

Electroretinographic investigation of ARSACS patients has been reported in only a few patients. Interestingly, one of six patients from the original publication and another single case exhibited full-field ERG abnormalities, but both were incidental findings in the absence of visual complaints (2, 6). Neither precise description nor the ERG waveforms were published. Results of OCT in patients with ARSACS have never demonstrated abnormalities or thinning of the outer retina. The present patient showed inner retinal dysfunction in both rod and cone systems, probably confined to the On− bipolar cells (Off− responses were normal). There are many causes of a “negative ERG” waveform, such as X-linked retinoschisis and forms of congenital stationary night blindness [e.g., Ref. (14)], but those disorders are usually symptomatic, whereas in this patient his only visual symptoms related to the keratoconus.

More than 100 mutations reported to date in ARSACS result in a decreased production of sacsin, a chaperone protein (15). Pathological studies in humans have revealed swelling both of thalamic and cerebellar neurons and, in a knockout mouse model of ARSACS, altered mitochondrial transport, slowing of axoplasmic flow within long axons, and an abnormal cerebellar dendritic network have been demonstrated (5, 15). Retinal examination reveals structural changes in the retina in most patients, particularly peripapillary RNFL thickening. Recently, it has been hypothesized that peripapillary RNFL thickening might result from the slowing of axoplasmic flow within retinal ganglion cells axons and that ARSACS may be a storage disorder (5). Accumulation of proteins at an inner retinal level, or a secondary effect on retinal bipolar cells, could explain the electroretinographic abnormalities described herein.

The electroretinographic data in this patient show that ARSACS can manifest inner retinal dysfunction in both rod and cone systems. Full-field ERG in a greater number of ARSACS patients would help determine the incidence of this dysfunction and further elucidate the nature and significance of the findings.

## Ethics Statement

Retrospective case studies (up to three cases) are exempted from being examined by the regional ethics committee of the Canton de Vaud [Commission Cantonale d’Éthique de la Recherche sur l’Humain (VD)]. Commission Cantonale d’Éthique de la Recherche sur l’Humain (VD)—extrait des lignes directrices quant à l’application de la LRH (Loi relative à la recherche sur l’être humain, entrée en vigueur dès Janvier 2014) (http://www.cer-vd.ch/soumission/premiers-pas.html).

## Author Contributions

F-XB: examined the patient and wrote up the manuscript; GH and FB: examined the patient and revised the manuscript.

## Conflict of Interest Statement

The authors declare that the research was conducted in the absence of any commercial or financial relationships that could be construed as a potential conflict of interest.
